# Subway Gearbox Fault Diagnosis Algorithm Based on Adaptive Spline Impact Suppression

**DOI:** 10.3390/e23060660

**Published:** 2021-05-25

**Authors:** Zhongshuo Hu, Jianwei Yang, Dechen Yao, Jinhai Wang, Yongliang Bai

**Affiliations:** 1 School of Mechanical-Electronic and Vehicle Engineering, Beijing University of Civil Engineering and Architecture, Beijing 100044, China; huzhongshuo@163.com (Z.H.); yaodechen@bucea.edu.cn (D.Y.); wangjinhai@bucea.edu.cn (J.W.); 2 Beijing Key Laboratory of Performance Guarantee on Urban Rail Transit Vehicles, Beijing University of Civil Engineering and Architecture, Beijing 100044, China; yongliangbai@126.com; 3 School of Mechanical, Electronic and Control Engineering, Beijing Jiaotong University, Beijing 100044, China

**Keywords:** gearbox, signal interception, peak extraction, cubic spline interpolation envelope

## Abstract

In the signal processing of real subway vehicles, impacts between wheelsets and rail joint gaps have significant negative effects on the spectrum. This introduces great difficulties for the fault diagnosis of gearboxes. To solve this problem, this paper proposes an adaptive time-domain signal segmentation method that envelopes the original signal using a cubic spline interpolation. The peak values of the rail joint gap impacts are extracted to realize the adaptive segmentation of gearbox fault signals when the vehicle was moving at a uniform speed. A long-time and unsteady signal affected by wheel–rail impacts is segmented into multiple short-term, steady-state signals, which can suppress the high amplitude of the shock response signal. Finally, on this basis, multiple short-term sample signals are analyzed by time- and frequency-domain analyses and compared with the nonfaulty results. The results showed that the method can efficiently suppress the high-amplitude components of subway gearbox vibration signals and effectively extract the characteristics of weak faults due to uniform wear of the gearbox in the time and frequency domains. This provides reference value for the gearbox fault diagnosis in engineering practice.

## 1. Introduction

The gearbox is an important part of an urban rail train, and a malfunctioning gearbox will significantly affect the smoothness and comfort of the train operation. Furthermore, the service life of the whole structure will be reduced due to the long-term wear of gear damage [1]. At present, in actual train operation management and maintenance, most of the gearbox fault diagnosis is completed during the maintenance of the unit. To find a gearbox fault as early as possible, the real vehicle signals must be processed and diagnosed [2]. However, for the signal processing of a real vehicle gearbox, the working environment of the gearbox is complex and easily affected by vibrations of all kinds of connected parts. This can lead to various problems, such as spectrum distortion caused by the impact of the rail joint gap, the failure frequency not being obvious due to the uniform wear of the gearbox, and the speed of the train and frequency of gearbox being in an uncertain state. The traditional signal processing methods have difficulty capturing the fault characteristics of a gearbox. Therefore, filtering the interference in a real vehicle signal and accurately extracting the fault characteristic information from the vibration time-domain signal are the key problems to be solved in the fault diagnosis of subway gearboxes.

In recent years, scholars have proposed a series of methods for processing the fault signals of nonstationary stochastic processes, and many algorithms and processing ideas have been improved and applied to gearbox fault diagnosis. As a previously proposed signal decomposition method, empirical mode decomposition (EMD) [3], has been gradually developed in recent years, the optimized form of EMD is now relatively mature and widely used in fault diagnosis [4]. Ensemble empirical mode decomposition (EEMD) has been proposed to reduce the mode mixing phenomenon [5,6,7,8,9,10] in the process of EMD decomposition. This is realized by adding Gaussian white noise to change the characteristics of the extrema, and variants of this algorithm have been developed. Wu et al. [11] proposed a feature learning detection method based on EEMD and a Gaussian process classifier. Trelet was used for data dimension reduction as the Gaussian process input, and it is optimized by bacterial foraging optimization. In addition, complementary set empirical mode decomposition (CEEMD) introduces complementary noise [12,13,14], which eliminates redundant noise to a large extent in the reconstruction of signals, greatly shortening the processing time and improving the computational efficiency. Han et al. [15] used a combination of the Teager energy operator and CEEMD to extract features from the bearing fault signals of a wind turbine. This algorithm showed unique advantages in detecting the impact characteristics of signals and effectively extracted the fault features of low-speed bearings. Li et al. [16] used the improved complete CEEMD with adaptive noise method to decompose bearing vibration signals, extracted nonlinear entropy features, and built a multiclass intelligent recognition model based on an integrated support vector machine, effectively classifying experimental data under various operating conditions. Variational mode decomposition (VMD) is a kind of adaptive signal decomposition method. This method assumes that each mode is around a center frequency. The modal solution can be converted to a constrained optimization problem, drastically reducing the modal aliasing phenomenon [17]. Based on this method, many scholars modified the VMD algorithm for mechanical fault diagnosis [18,19,20,21,22]. Cai et al. [23] proposed a multipoint kurtosis–VMD compound algorithm that can reduce the original signal noise and extract recurrent failures by using multipoint kurtosis at the same time, and more cycle vectors were constructed to determine the decomposition layers K. Compared with the traditional particle swarm optimization algorithm and ant colony algorithm, this method shows a speed advantage and eventually effectively extracts the compound fault characteristics. Hua et al. [24] proposed an inherent mode function selection method based on the resonance frequency for VMD parameter optimization and selected the modal components with fault information according to the resonance frequency. The results showed that this method can extract weak signals of early rolling bearing faults and realize correct judgment of bearing faults. Liu et al. [25] improved the VMD algorithm based on an autoregression (AR) model. By reducing the interference of low-frequency components and denoising each component, Hilbert envelope analysis was used to demodulate the signal, and the fault was determined by combining the demodulation frequency and theoretical fault frequency. Miao et al. [26] proposed an improved parameter-adaptive VMD method to construct a new comprehensive kurtosis index, which takes into account periodicity and impacts. This algorithm is superior to the traditional VMD method and further extends the application of VMD for compound fault diagnosis. Many scholars have also applied neural networks for fault diagnosis [27,28,29,30,31,32,33,34,35], and the improved algorithms have shown good results. In addition, there are also many decomposition methods based on signal processing, including local mean decomposition [36,37,38,39,40], Fourier decomposition [41], and other methods, which are widely used in gearbox fault diagnosis. Lei et al. [42] combined the advantages of integrated local mean decomposition and fast spectral kurtosis to carry out fault detection of rotating machinery and finally verified the effectiveness of this algorithm for the fault diagnosis of gearboxes and rolling bearings. Dou et al. [43] proposed a mechanical fault feature extraction method based on Fourier decomposition, which has the characteristics of adaptive narrowband filtering at high and low frequencies. In terms of separating low-frequency signal components, due to the traditional EMD method, it has a good effect when used for the feature extraction of mechanical vibration signals. Pang et al. [44] proposed an enhanced singular spectrum decomposition (ESSD) method that highlights fault signal components by introducing differentiation and integration operators. This method exhibits strong anti-interference abilities and better performances when processing experimental signals compared with traditional singular spectrum decomposition (SSD) and VMD.

For the research on wheel–rail impact caused by rail joints, Yang et al. [45] designed an experiment and finite element model to reproduce the noise and vibration signals of a wheelset passing the rail joints. The results show that the vibration energy of the vertical impact is mainly concentrated around 300 and 1000 Hz, while the dominant frequency range of transverse vibration is between 300 and 1200 Hz. Tajalli et al. [46] used an experiment and finite element model to study different relative positions of sleeper and rail joint influence on wheel–rail impact; the results show that the wheel–rail contact force is 1.77 times the size of the static load when wheelset passes the rail joint, which caused the wheel–rail vibration acceleration signal to change sharply. The research also points out that the impact force can be reduced by 40% when using a fishplate to connect the rail joint. Choi et al. [47] used the ANSYS model to analyze the wheel–rail impact force at the rail joint, and the model was verified by experimental data. They evaluated the wheel–rail contact force by comparing track impact factor (TIF), and the results showed that TIF value with rail joints is 57% higher than that for continuous welded rails. This research shows that rail joints produce wheel–rail impact, which will bring interference components to gearbox diagnosis. Real vehicle signal data are very scarce currently, and this problem is proposed in real vehicle signal processing. The traditional EMD and VMD methods have remarkable performance on the bearing vibration signals collected in the laboratory, but the effect on the wheel–rail impact filtering in the measured train gearbox signals is not ideal; most of these algorithms were proposed based on laboratory data, and their feasibility in practical engineering needs to be further verified. The method proposed in this paper aims to eliminate the interference of wheel–rail impact on the spectrum more effectively.

To solve this problem, this paper proposes a time-domain adaptive interception algorithm based on a cubic spline interpolation envelope. The purpose is to remove wheel–rail impact interference in real train signals. The basic idea is to automatically extract the peak value from the whole large sample signal and calculate the train speed data corresponding to the short-term sample by using the peak interval. Based on the position of the peak value, the single large sample signal is adaptively intercepted and divided into several short-term samples, after which it is analyzed in the time–frequency domain and finally compared with healthy signals. The results show that this method can effectively eliminate the low-frequency interference caused by rail impacts, and it can calculate and complement the missing train speed data. Moreover, the processed signals can reflect certain fault rules in both the time-domain statistics and frequency-domain analysis, and the method can diagnose uniform wear of subway gearboxes.

The main contributions of this work are summarized as follows: A gearbox fault diagnosis algorithm based on adaptive spline is proposed for suppressing impact components in real vehicle signals adaptively. Speed data can also be calculated based on the proposed algorithm. A new feature representation method is used in the analysis part; it is based on multisample preponderance after processing by the proposed algorithm.

This work has the following organization: The foundational spline method and the proposed algorithm are both introduced in Section 2. Section 3 describes the acquisition of real vehicle signal and presents the results of the analysis of the proposed method carried out using real vehicle signal, compared with EEMD and VMD. Conclusions are drawn in Section 4.

## 2. Signal Segmentation Method Based on Cubic Spline Interpolation Envelope

The impact components will seriously interfere with the low frequency after Fourier transform, which causes severe distortion of the spectrum. At present, there may be some difficulties in finding the peak and intercept signal adaptively. In this section, a time-domain adaptive interception algorithm based on cubic spline interpolation envelope is proposed to deal with interference of wheel–rail impact in real vehicle signals. A long-time large sample signal is divided into several short-term small sample signals, and impact components are extracted and eliminated at the same time.

### 2.1. Cubic Spline Interpolation Envelope

The cubic spline interpolation method has good convergence properties. In the process of adaptive signal segmentation, it exhibits good stability. Cubic spline interpolation uses multiple piecewise cubic polynomials and is piecewise continuous, so its first and second derivative functions are continuous, and there are no errors at the nodes or at the nodes of the first and second derivatives.

Cubic spline interpolation is defined as a partition $x_{0}<x_{1}<$ … $<x_{n-1}<x_{n}$ given in the interval $\left[x_{0},x_{n}\right]$. The function L(x) is defined on each segment interval $\left[x_{i-1},x_{i}](i=1,2,\ldots ,n\right)$. L(x) is a cubic polynomial, and in the entire interval $\left[x_{0},x_{n}\right]$, L(x) is a second-order continuous and differentiable function. Each polynomial satisfies the following at each node $x_{i}(i=1,2,\ldots ,n-1)$:

(1) $$L^{\left(k\right)}(x_{i}-0)=L^{\left(k\right)}(x_{i}+0),k=0,1,2,$$

The constraint conditions are as follows:

(2) $$\left\{\begin{array}{l} L(x_{i})=y_{i},(i=0,1,\ldots ,n)\\ L(x_{i}-0)=L(x_{i}+0),(i=1,2,\ldots ,n-1)\\ L{}^{\prime }(x_{i}-0)=L{}^{\prime }(x_{i}+0),(i=1,2,\ldots ,n-1)\\ L{}^{\prime \prime }(x_{i}-0)=L{}^{\prime \prime }(x_{i}+0),(i=1,2,\ldots ,n-1) \end{array}\right.,$$

In the cubic spline interpolation method, there are three types of boundary conditions: clamped, natural, and not-a-knot. Since the second derivative of cubic polynomial L(x) is a first-order function, let $\left[L{}^{\prime \prime }(x_{0}),L{}^{\prime \prime }(x_{1}),\ldots ,L{}^{\prime \prime }(x_{n})]=[Q_{0},Q_{1},\ldots ,Q_{n}\right]$, and then the interpolation function of each piecewise interval is as follows:

(3) $$\begin{array}{c} L(x)=\frac{Q_{i}-1}{6h_{{}_{i}}}{\left(x_{i}-x\right)}^{3}+\frac{Q_{i}}{6h_{i}}{\left(x-x_{i-1}\right)}^{3}+(\frac{y_{i}-y_{i-1}}{h_{i}}-\frac{Q_{i}-Q_{i-1}}{6}h_{i})x\\ +y_{i}-\frac{Q_{i}}{6}h_{i}{}^{2}-(\frac{y_{i}-y_{i-1}}{h_{i}}-\frac{Q_{i}-Q_{i-1}}{6}h_{i})x_{i} \end{array}$$

In Equation (3), $h_{i}=x_{i}-x_{i-1}$.

**Figure 1:**
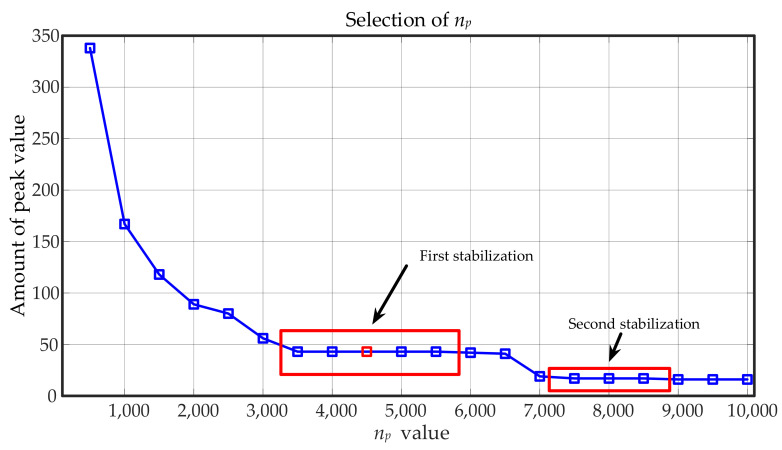
The selection of $n_{p}$ value.

With the support of the above principle, the original signal is processed by a cubic spline interpolation envelope. In the whole signal, the amplitude sequence is x, the corresponding time series is t, and n+1 signal sequence points are denoted as $\left(t_{0},x_{0}\right)$, $\left(t_{1},x_{1})\ldots (t_{i},x_{i})\ldots (t_{n},x_{n}\right)$. The step size to find the maximum number of points $n_{p}$ is defined, and the maximum value from the fixed signal step size is output. The signal length is optimized based on experience. An excessive step size will cause significant distortion, while too small of a step size will increase the number of calculations. Therefore, it is necessary to optimize the selection of step size, as shown in Figure 1, which is the amount of peak value under different step sizes. It can be seen that when the step size reaches a certain range, the amount of peak value tends to be stable several times. In the first stabilization, the average of corresponding values will be the best $n_{p}$ (red mark), and then the coordinate of each maximum point is $(a_{j},b_{j}),j=0,\ldots ,n$.

Using the maximum output point $\left(a_{j},b_{j}\right)$ in the above fixed step size $n_{p}$ as the interpolation point, Equation (3) shows that the cubic spline interpolation function on the interval $\left[a_{j},a_{j+1}\right]$ is expressed as follows:

(4) $$\begin{array}{c} L(t)=\frac{Q_{j}-1}{6h_{{}_{j}}}{\left(a_{j}-t\right)}^{3}+\frac{Q_{j}}{6h_{j}}{\left(t-a_{j-1}\right)}^{3}+(\frac{b_{j}-b_{j-1}}{h_{j}}-\frac{Q_{j}-Q_{j-1}}{6}h_{j})t\\ +b_{j}-\frac{Q_{j}}{6}h_{j}{}^{2}-(\frac{b_{j}-b_{j-1}}{h_{j}}-\frac{Q_{j}-Q_{j-1}}{6}h_{j})a_{j} \end{array}$$

In Equation (4), $h_{j}=a_{j}-a_{j-1}$. A fixed boundary condition is selected; that is, the first derivative value at each maximum coordinate point $a_{0}$ and $a_{j}$ is $L{}^{\prime }(a_{0})=b{{}^{\prime }}_{0}=L{}^{\prime }(a_{n})=b{{}^{\prime }}_{n}=0$, and the solution of $Q_{j}$ is as follows:

(5) $$\left[\begin{array}{ccccc} 2 & 1 & & & \\ \mu_{2} & 2 & 1-\mu_{2} & & \\ & \ddots & \ddots & \ddots & \\ & & \mu_{n-1} & 2 & 1-\mu_{n-1}\\ & & & 1 & 2 \end{array}\right]\left[\begin{array}{c} Q_{1}\\ Q_{2}\\ \vdots \\ Q_{n-1}\\ Q_{n} \end{array}\right]=\left[\begin{array}{c} \lambda_{1}\\ \lambda_{2}\\ \vdots \\ \lambda_{n-1}\\ \lambda_{n} \end{array}\right],$$

where $\mu_{j}=\frac{h_{j}}{h_{j}+h_{j+1}}$, $\lambda_{1}=\frac{6}{h_{1}}(\frac{b_{1}-b_{0}}{h_{1}}-b{{}^{\prime }}_{0})$, $\lambda_{n}=\frac{6}{h_{n}}(b{{}^{\prime }}_{n}-\frac{b_{n}-b_{n-1}}{h_{n}})$, and $\lambda_{j}=6(\frac{b_{j+1}-b_{j}}{h_{j+1}}-\frac{b_{j}-b_{j-1}}{h_{j}})\frac{1}{h_{j}+h_{j+1}}(j=2,3,\ldots ,n-1)$.

Through the catch-up method, $\left[Q_{0},Q_{1},\ldots ,Q_{n}\right]$ can be solved for using Equation (5), which can be back-substituted into Equation (5) to obtain the cubic spline interpolation envelope of the original signal. At this time, the envelope curve has filtered out a significant amount of peak interference other than the wheel–rail impact, and the maximum value of the envelope curve can be determined and output as $\left(G_{i},H_{i}\right)$, i=0,1,…,n.

### 2.2. Impact Component Extraction and Short-Term Signal Sample Segmentation

There is some error between the crude extracted coordinates and the original signal due to the envelope curve fitting. Therefore, to eliminate this error, the original signal peak is extracted. Because the time interval between the wheel and rail impact has a certain regularity, that is, the ratio of the rail length to vehicle speed, the abscess interval of the pulse can be estimated as Δt ≈ 1.4 s. The search interval $\left[G_{i}-\frac{\Delta t}{2}•f_{s},G_{i}+\frac{\Delta t}{2}•f_{s}\right]$ is set according to the abscissa of the maximum value of the envelope curve $G_{i}$, and the maximum values $\left(G_{i}{}^{\prime },H_{i}\prime \right)$, i=0,1,…,n are found within the search interval to complete the extraction steps of the whole signal.

After the impact component is extracted, the x-coordinate difference of maximum values $\left(G_{i}{}^{\prime },H_{i}{}^{\prime }\right)$, which is $\Delta G=G_{i}{}^{\prime }-G_{i-1}{}^{\prime }$, is calculated; ΔG is the interval time of the train passing along a steel rail. To control the length of each short-term sample and reduce the number of variables, a 1 s signal is intercepted between the extracted wheel–rail impact interval to complete the overall signal segmentation.

## 3. Experiment and Results

### 3.1. Real Vehicle Data Collection

The vibration acceleration data of a real vehicle gearbox were collected. Test information is as follows:

Data acquisition equipment: three-axis vibration sensor (measurement range is 500 g), MDR-80 dynamic acquisition instrument, MDR-80 mobile data recording system.

Test object: subway vehicle.

Test location: a whole Beijing subway line, total distance of single acquisition is about 81 km.

**Figure 2:**
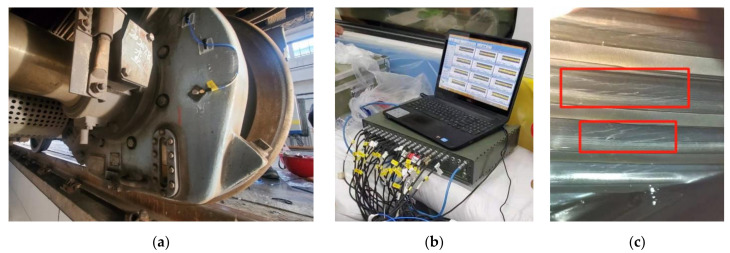
Data acquisition of real vehicle gearbox: (**a**) gearbox sensor layout; (**b**) data acquisition equipment; (**c**) tooth surface fault.

Figure 2a shows the field sensor layout, and the vibration sensor was installed on the outer shell of the gearbox. The sensor adopted a triaxial vibration sensor, and the sampling frequency was set to 10 kHz. Figure 2b shows the data acquisition equipment. The acceleration and deceleration processes of the vehicle were kept as uniform as possible. When driving at a constant speed on a straight line segment, the speed should be kept at about 67 km/h, and the stopping time of each stop should be 2 s. The data of the faulty and healthy gearbox were collected on the same subway line twice. Two data samples were collected, and the single sample collection time lasted about 120 min. The total number of sample points was above 70 million for one sample. The tooth ratio of the measured gearbox was 100:13. The surface damage of the internal gear of the fault gearbox is shown in Figure 2c, and there were cracks, peeling, and wear on the gear surface. The other calculation parameters are shown in Table 1.

### 3.2. Results

**Figure 3:**
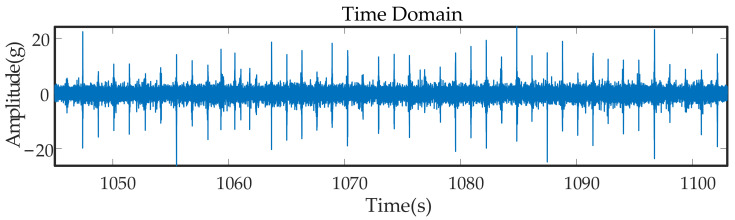
Time-domain diagram of original signals.

Figure 3 shows a segment of the signal when the vehicle was operating at a uniform speed in the time domain. Wheel–rail impacts were distributed uniformly over the whole segment of the time-domain signal. After the Fourier transform, the spectral components were mixed, and the fault components could not be determined and separated. The fault frequency could not be distinguished, and there was more interference at low frequencies, which was caused to some extent by the frequency of rail joint gap impacts.

**Figure 4:**
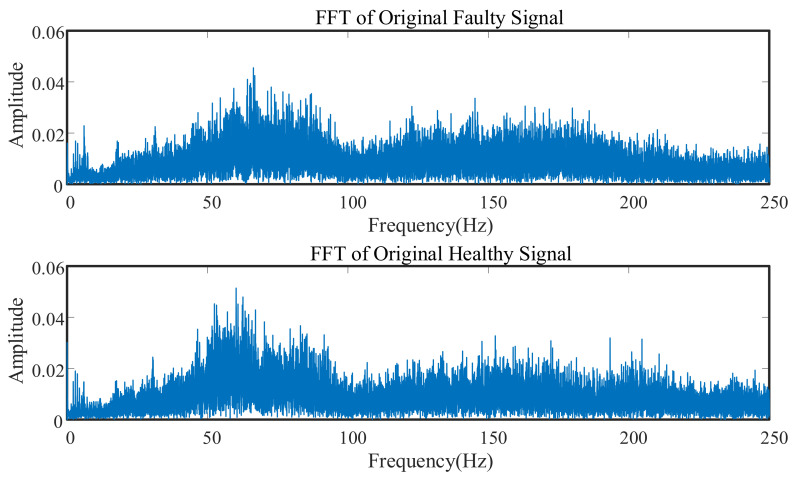
Spectrum comparison of healthy and faulty states.

Tooth surface wear is an inevitable phenomenon over the life of a gearbox. The failure frequency was not fixed, the train speed varied during the signal measurement, and the gear switching frequency floated up and down. Figure 4 shows the healthy and faulty original signal spectrum graph. The original noise signal interference was evident, and various frequency components were mutually coupled. The signal characteristics of some weak faults were easily submerged by noise, so it was difficult to judge the fault situation based on the spectrum peaks. 

**Figure 5:**
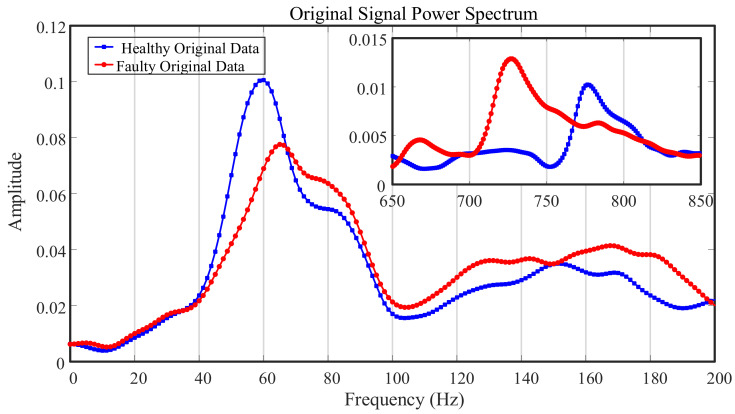
Power spectrum of original signal.

Figure 5 shows the original signal power spectrum diagram, and the inset shows the meshing frequencies. The red line represents the faulty signal, and the blue line represents the healthy signal. The healthy and faulty states of the power spectrum in the frequency domain were similar, and the power of the healthy signal was even greater than that of the faulty signal in one region. A pattern of the differences between the two spectra is not evident, and the power spectrum analysis of the original signal cannot show the fault characteristics.

Based on the principle described above, the cubic spline interpolation envelope signal intercept method was applied. The real vehicle operating conditions and changes interfered with the signal, and the gearbox signal was mixed with the wheel–rail impact signal. Thus, direct calculation and analysis of the original signal could not provide better analysis of the composition of the gearbox fault.

**Figure 6:**
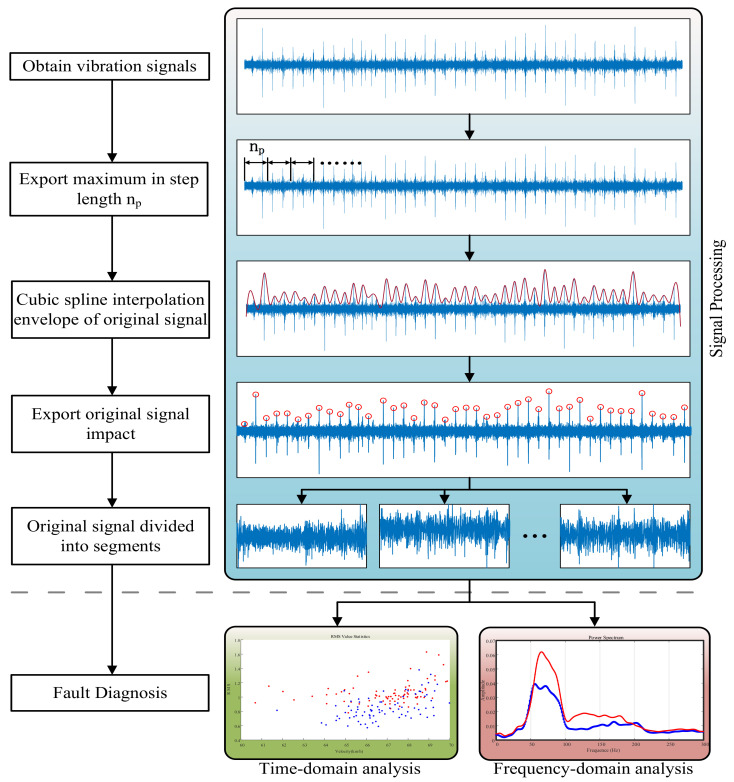
Algorithm flow chart.

The specific steps of the algorithm are as follows, and the algorithm flow chart is shown in Figure 6:

(1) The extremum step $n_{p}$ is defined to find and output the maximum value $\left(a_{j},b_{j}\right)$ from the extremum step in the original signal.

(2) Using the cubic spline interpolation method, the envelope curve S(t) is calculated for the above maximum array, and the maximum value $\left(G_{i},H_{i}\right)$ of the envelope is output.

(3) The maximum value $\left(G_{i}{}^{\prime },H_{i}{}^{\prime }\right)$ of the original signal is found within half of the abscess interval of the maximum value, which is the peak value of the original signal rail joint gap impact.

(4) According to the coordinates of the adjacent impact peak, some of the short-term sample signals between the peak values were intercepted, and a large number of short-term sample signals were intercepted for statistical analysis in the time–frequency domain.

**Figure 7:**
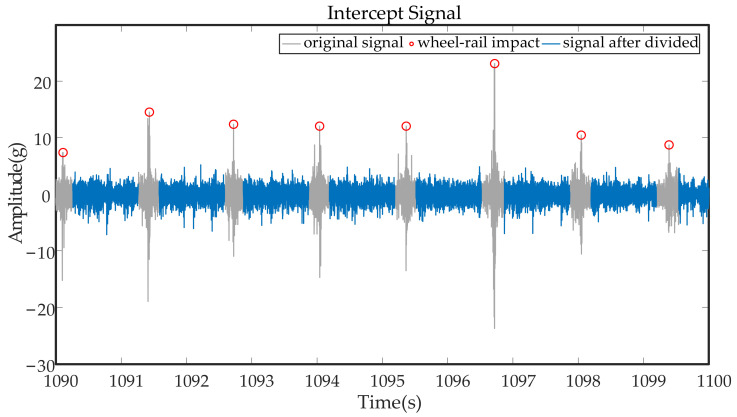
Schematic diagram of signal interception.

In this way, the wheel–rail impact components in the original signal can be effectively removed, and the sample size of the data can also be increased, laying a foundation for the next step of the time-domain statistics. Figure 7 shows the whole signal segment. The signal after adaptive interception is shown, where the gray components are the original signal and the blue components are parts of the intercepted signal. The rail joint gap impact component was effectively eliminated, and a gearbox signal component with a more uniform amplitude was retained. Wheel–rail impact components are marked in red circles; their accuracy directly determines the effectiveness of the algorithm. After many instances of verification and calculation, the accuracy rate was about 98%, and errors often occurred at the boundary part of signals.

**Figure 8:**
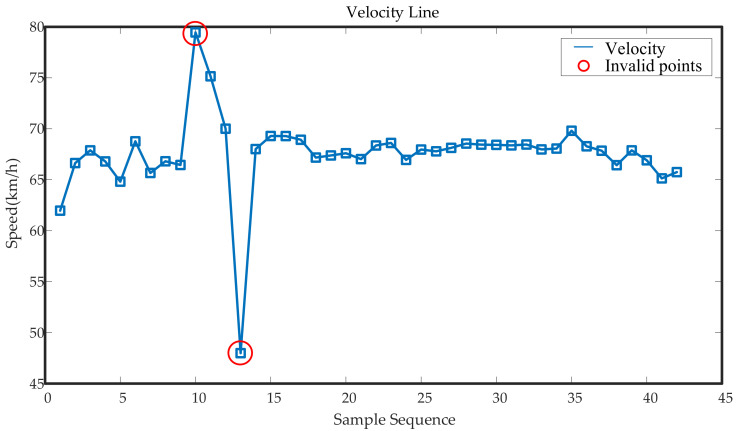
Velocity line diagram.

Since the original measured signals often lack the speed information of the train when it is operating, the calculation of the speed data is completed according to the extracted time between the wheel–rail impact peak and the length of the rail. The parameters such as the length of the rail and the measured wheel diameter are shown in Table 1, and the train speed is $v=\frac{l_{r}}{\Delta G}$. It is also possible to calculate the pinion frequency $f_{r}=\frac{v}{\pi d}$, the large gear frequency $f_{R}=f_{r}•i$, and the meshing frequency $f_{m}=100f_{r}=13f_{R}$. It should be noted that the speed calculated at this time is the average speed of the train passing a whole section of rail, as shown in Figure 8. After the signal interception, there are some incorrect calculated speed data. Due to changes in speed and rail length, the distance between impacts may become irregular, which will make calculated speed data invalid. To ensure that the data were collected when the train moved at as constant a speed as possible, the calculated speed was used as the discriminant standard. Outlying speed points were screened out by setting a threshold: the maximum acceleration could not be greater than 1.5 m/s^2^ when the train was running. In the figure, the slope of the curve pointed to by the arrow is too steep, so points on either side of this point will be regarded as outlying speed data, and short-term samples on either side of the point will not be analyzed in the time–frequency domain. At this point, the entire adaptive signal segmentation processing was completed.

### 3.3. Statistical Analysis of Time-Domain Root Mean Square Values

**Figure 9:**
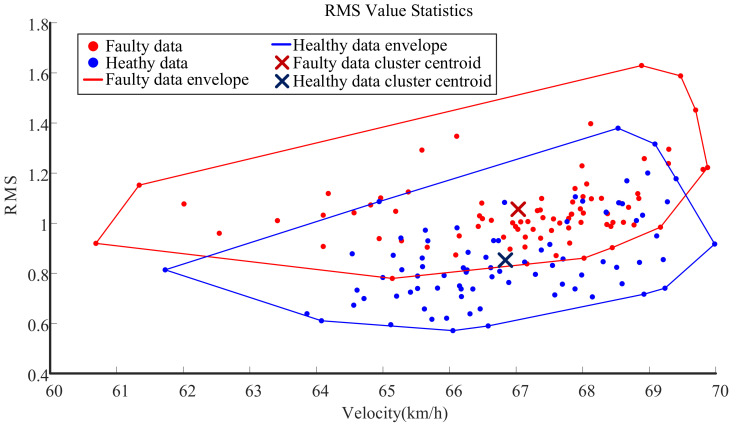
Statistics on root mean square values of short-term samples.

First, the state of the gearbox was judged in the time-domain analysis. Since a single large sample was divided into several short-term samples after signal segmentation, the time-domain statistical analysis was carried out with the advantage of multiple samples, and the main calculated parameter was the root mean square (RMS) value. The time-domain index RMS of each signal after segmentation was calculated. The RMS is plotted versus the train speed for both the healthy and faulty states in Figure 9. The blue points are the healthy state data, the red points are the faulty state data, the red line is the faulty data envelope, the blue line is the healthy data envelope, and the dark red and dark blue X marks are the faulty and healthy data scatter centers of mass, respectively. From the perspective of the root mean square value, the overall level and average level of the faulty state were 30% higher than those of the healthy state. Combined with the speed distribution, although the root mean square value from low speed to high speed had a slight linear increase, the faulty state level was always above the healthy state.

**Figure 10:**
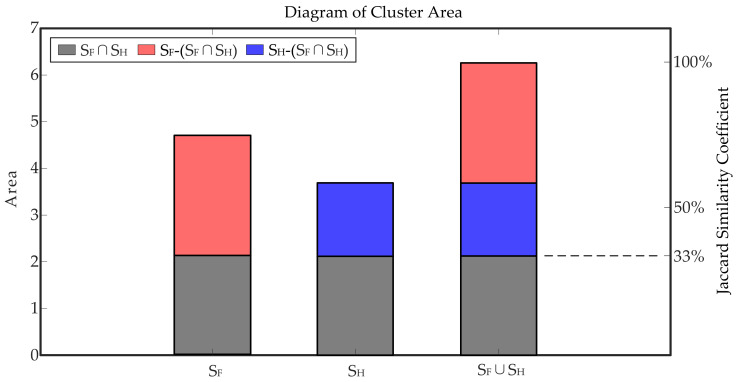
Cluster area and Jaccard similarity coefficient.

The Jaccard similarity coefficient of the two types of cluster areas is used to indicate the similarity degree of the clusters. The formula of the Jaccard coefficient is as follows: $J(S_{H},S_{F})=\frac{\left|S_{H}\cap S_{F}\right|}{\left|S_{H}\cup S_{F}\right|}$, where $S_{H}$ is the area of the convex envelope of the healthy scattered points and $S_{F}$ is the area of the convex envelope of the faulty scattered points. As shown in Figure 10, the Jaccard similarity coefficient of the two clusters was 33.87%, indicating that the similarity degree of the two types of data was extremely low. This further indicated that there was a significant difference between the healthy and faulty data, and the fault features of the time-domain root mean square statistics were prominent.

### 3.4. Multiple Mean Power Spectrum Analysis in Frequency Domain

After the fault differences were found in the time domain, the fault characteristics were further characterized in the frequency domain. According to the above analysis, since the train speed varied continuously, the average speed of the short-term sample train was 66 km/h. To determine the rotational frequency range of the large and small gears, the rotational frequencies of the small and large gears were 55.5 and 7.22 Hz, which were calculated using the wheel diameter and tooth ratio.

**Figure 11:**
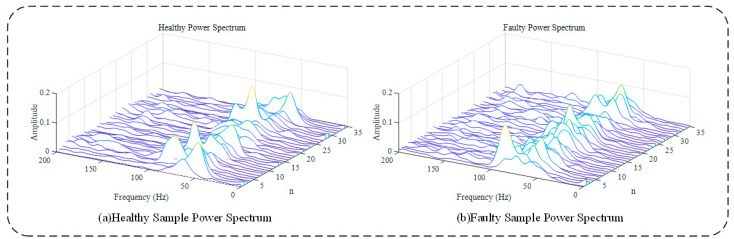
Power spectrum of each short-term sample.

The signal segmentation algorithm proposed in this paper was used to divide the large sample signal into several short-term sample signals, and then the power spectrum of each small sample signal was calculated. To control the variables as much as possible, 35 short-term healthy samples and 35 short-term faulty samples were selected, and all the samples were collected from the same section on the rail line. The signal power spectrum is shown in Figure 11, where each curve represents the signal power spectral density for a short-term sample. Figure 11a,b show the results for healthy and faulty gearboxes, respectively. Most of the peak values of the faulty sample were concentrated at small gear rotation frequencies. Relative to the healthy data, the curve of the faulty data was steeper, and the peak frequency range was more concentrated. However, the above characteristics were not prominent in the large gear rotational frequency range.

**Figure 12:**
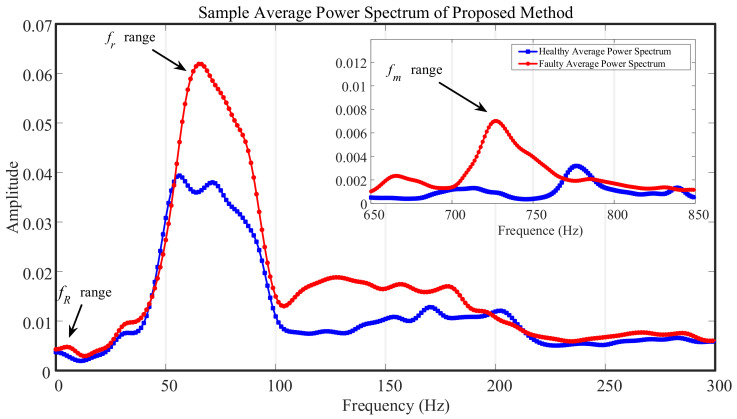
Average power spectrum of short-term samples with proposed method.

To represent the differences in the characteristics, the average power spectra of the faulty and healthy states were obtained, as shown in Figure 12. The main plot shows the power spectrum of the gear rotational frequency, and the inset shows the power spectrum of the meshing frequency. The blue line represents the healthy state, and the red line represents the faulty state. The power spectral density function of the fault near the gear rotational frequency was much higher than that of the healthy state, especially in the region corresponding to the double small gear rotational frequency. Due to cracks and spalling failures in the gear surface and the changing speed of the train, the frequency-domain features had no specific fault frequency, which confirmed the fault features of multiple cracks and wear on the gearbox tooth surface. Thus, the fault diagnosis of the measured gearbox data was complete.

**Figure 13:**
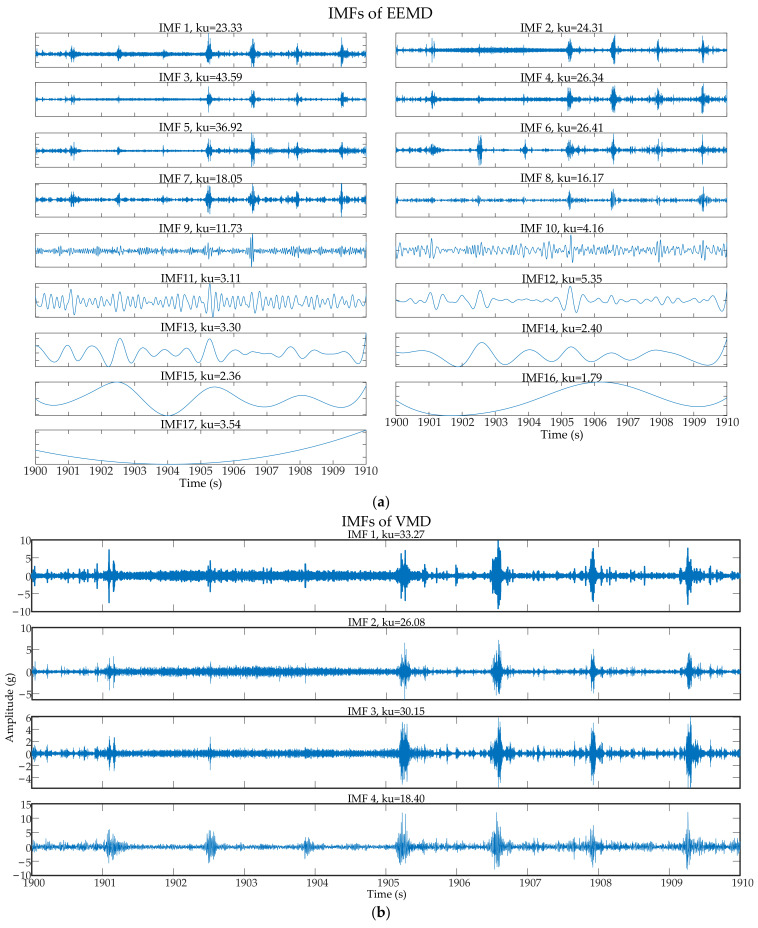
IMFs after decomposition by EEMD and VMD: (**a**) IMFs of EEMD; (**b**) IMFs of VMD.

**Figure 14:**
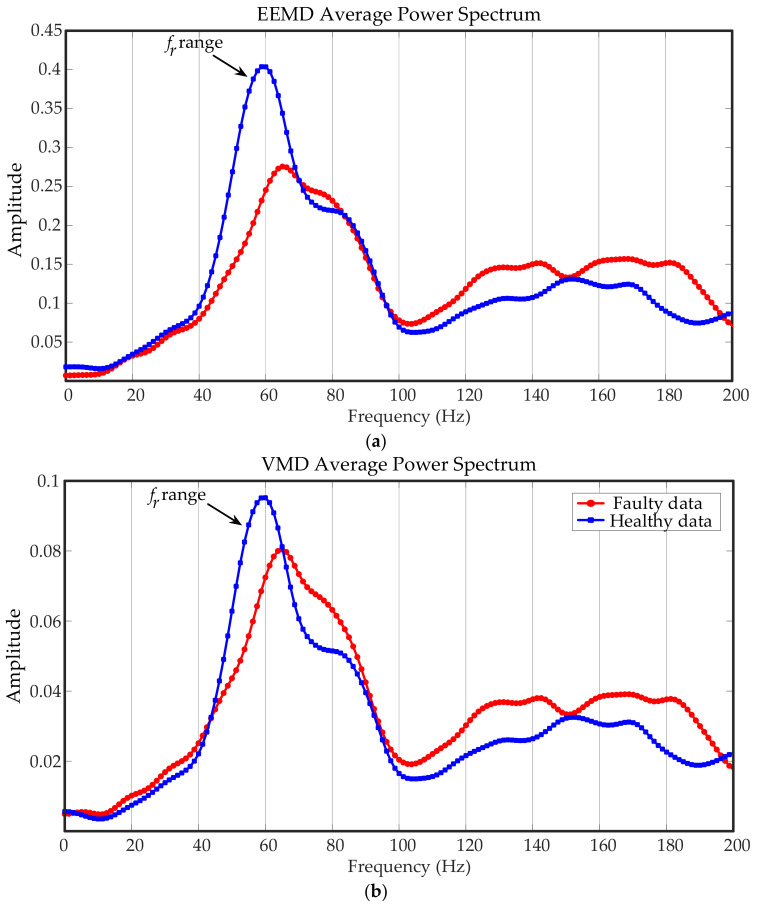
Average power spectra of compared methods: (**a**) EEMD average power spectrum; (**b**) VMD average power spectrum.

EEMD and VMD were selected to be compared with the proposed method, and the signal was divided into several 10 s short-term signals. After decomposed by using the two methods, IMFs were obtained, as shown in Figure 13. The IMFs with larger kurtosis were selected for reconstruction. The power spectra of reconstructed signals are shown in Figure 14. It can be clearly seen that EEMD and VMD failed to filter the impact components, which still affected the spectra significantly.

**Figure 15:**
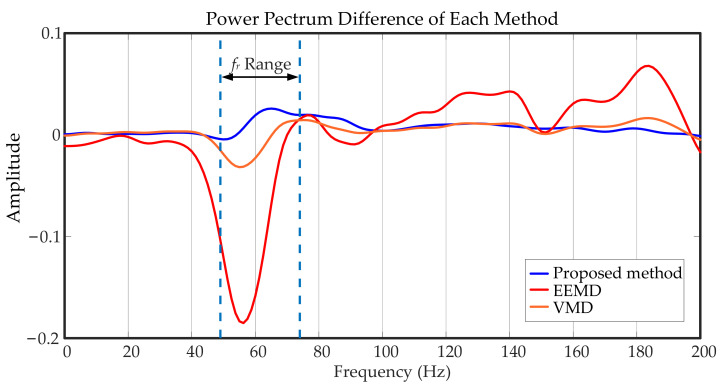
Power spectrum difference of each method.

The difference between healthy and faulty power spectra was calculated to evaluate the methods’ effects. As shown in Figure 15, a larger value at the $f_{r}$ range means the method has a better effect. It can be seen that the value of EEMD is far less than 0, and VMD is slightly better than EEMD, but it is still in the state of less than 0, which means it failed to distinguish between faulty and healthy signals. The method proposed in this paper has the best effect, with a value that is much greater than 0. In summary, removing the wheel–rail impact interference from the original signal can effectively highlight the gearbox fault features with cracks, wear, and other damage.

## 4. Conclusions

In this paper, measured signals of real trains were analyzed. This work provides reference value for the fault diagnosis of train gearboxes in engineering practice. A signal segmentation algorithm was proposed, and the advantages of this algorithm are as follows:

(1) The impacts between the wheelsets and rail joint gaps in a real vehicle signal cause significant spectrum distortion in the low-frequency region. However, the signal also exhibits a certain regularity in the time domain. By using this regularity, this paper proposes a segmentation algorithm based on cubic spline interpolation. It can suppress the high amplitude of the shock response signal and divide a single large sample signal into several short-term sample signals.

(2) In the case of an uncertain train speed, this method can calculate the data for the train speed by using the extracted wheel–rail impacts, which provides a certain data basis for the calculation of the rotational frequency and fault frequency band in the frequency-domain analysis.

(3) Taking advantage of the number of samples after signal segmentation, statistical analysis of the short-term sample signal was completed in the time and frequency domains. The results showed that the algorithm has a strong ability to filter the disturbances caused by the impact of the rail joint gap. It can extract the useful short-term signal sample accurately. Compared to EEMD and VMD, the proposed algorithm can suppress and remove the impact components of the original real vehicle signal effectively. The results can detect regularities in the faulty signal and highlight the gearbox fault characteristics due to cracks, wear, and other damage in the time- and frequency-domain analyses.

## Figures and Tables

**Table 1:** Train speed calculation parameters.

	$\mathrm{Length}\;\mathrm{of}\;\mathrm{Rail}/l_{r}(m)$	Wheel Diameter/d (mm)	Gear Ratio/i
**Parameter**	25	821	100:13

## Data Availability

Data sharing not applicable.
